# Synthesis and Characterization of Mesoporous Silica Functionalized with Calix[4]arene Derivatives

**DOI:** 10.3390/ijms131013726

**Published:** 2012-10-23

**Authors:** Sana M. Alahmadi, Sharifah Mohamad, Mohd Jamil Maah

**Affiliations:** 1 Department of Chemistry, Faculty of Science, University Malaya, Kuala Lumpur 50603, Malaysia; E-Mails: sharifahm@um.edu.my (S.M.); mjamil@um.edu.my (M.J.M.); 2 Department of Chemistry, Faculty of Science, Taibah University, Almadina Almonwara 30001, Saudi Arabia

**Keywords:** MCM-41, calix[4]arene, *p*-Sulfonatocalix[4]arene, para-*tert*-butyl calix[4]arene

## Abstract

This work reports a new method to covalently attach calix[4]arene derivatives onto MCM-41, using a diisocyanate as a linker. The modified mesoporous silicates were characterized by fourier transform infrared spectroscopy (FTIR), thermal analysis (TGA) and elemental analysis. The FTIR spectra and TGA analysis verified that the calix[4]arene derivates are covalently attached to the mesoporous silica. The preservation of the MCM-41 channel system was checked by X-ray diffraction and nitrogen adsorption analysis.

## 1. Introduction

Calixarenes is a flexible type of macrocyclic compounds that has soft π-donor cavities consisting of benzene rings along with hard oxygen cavities located on the hydroxyl lower rim. It is increasingly gaining importance in the field of host-guest chemistry owing to its formation of complexes with ions and neutral molecules [1–5]. More specifically, the functionalization of the lower rim calix[4]arenes coupled with appropriate binding groups resulted in sequences of significant cation receptors whose efficiency and selectivities were linked with both calixarenes ring size and conformation. In addition, calixararenes have been integrated into mobile phases or onto matrices to be considered as stable stages for gas chromatography (GC), capillary electrophoresis (CE) and liquid chromatography of high performance (HPLC) [6–9]. The attainments revealed that the ability of the macrocyles to recognize could significantly enhance most of the solutes separation selectivities, particularly aromatics [10,11]. The initial preparation of silica-bonded calix[4]arene tetraester was conducted by Glennon co-workers in 1993 [12–14] who utilized it in the separation of metal ions and amino acid esters in HPLC. Owing to their high surface areas and stable array of huge pore channels, MCM-41 materials are attractive. Moreover, the high concentration of silanol groups enables the creation of varying methods for covalent organic compound attachment. It is interesting to see what happens when the two kinds of molecules (Calixarenes and mesoporous silica) are combined. To the best of our knowledge, only few reports concerning this topic have been published to date using different silane coupling agents. Three of these works are the modification of mesoporous silica type SBA-15 with calix[4]arene derivatives using different silane coupling agents [15–17] and the last one is the MCM-41 mesoporous silica covalently bonded with rare earth complexes functionalized by two kinds of calixarenes using 3-(Isocyanatopropyl) triethoxysilane as a crosslinking agent [18]. Functionalization of mesoporous silica with deferent calix[4]arenes will lead to various materials with different selectivity for various guest ions and small molecules. Furthermore functionalization of mesoporous silica materials may have further application potential, such as in sensing materials [17] and solid phase extraction [15,16]. However, the synthesis of MCM-41 mesoporous materials covalently bonded with calix[4]arene derivatives using toluene isocyanate as linker has not been reported by other group up until now. Isocyanates are considered to highly react with –OH groups to produce urethane bonds [19,20]. Based on the used isocyanate, the binding strength to the surface of the support can be greater or at least equal to those obtained for the organosilane binders [20]. Taking into account the above we here present the systematic and comparative study of the MCM-41 mesoporous silica functionalized by calix[4]arene (C4), *p*-sulfonatocalix[4]arene (C4S) and *p*-*tert*-butyl-calix[4]arene (PC4) using toluene diisocyanate as a linker.

When using this method, one of the isocyanate endings is attached to the mesoporous silica surface, while the other remains available for reaction with the calix[4]arene.

## 2. Results and Discussion

In this study, three mesoporous silica modified with calix[4]arene derivatives have been prepared via modification of activated mesoporous silica with toluene 2,4-di-iso-cyanate (TDI) as linker and C4, C4S and PC4. Toluene 2,4-di-iso-cyanate was utilized to establish a bridge between the surface of mesoporous silica and calix[4]arene derivatives. TDI has highly unsaturated bonds and two isocyanate groups with different activities towards hydroxyl groups, located at a para-position and an ortho-position, respectively, and consequently, it is very active to hydroxyls. The isocyanate groups at para-positions would react with the hydroxyl groups on the surface of mesoporous silica preferentially whereas those at the ortho-positions would be preserved due to steric hindrance within the TDI molecule [21,22]. The mole amount of the isocyanate groups that reacted with mesoporous silica can be regarded as that of TDI that reacted with mesoporous silica. The amounts of TDI that reacted with mesoporous silica are largely dependent on the amount of hydroxyls on the mesoporous silica surface, so in the case of excess of TDI, the amounts of TDI that reacted with silica were invariable [23]. Excess TDI was used serving two functions; as a solvent to disperse silica and as a reactant in order to drive the reactions to completion, and it was easily removed after reaction by centrifugation and prolonged washing with anhydrous toluene. The isocyanate groups at ortho-positions in mesoporous silica-TDI reacted with hydroxyl groups at the lower rim of calix[4]arene derivatives to form modified mesoporous silica with toluene 2,4-di-iso-cyanate (TDI) as linker and C4, C4S and PC4.

**Figure 1:**
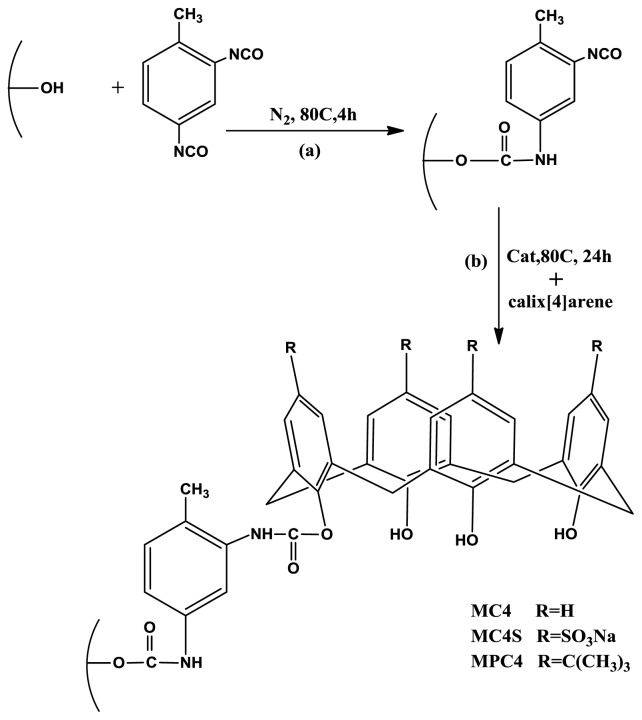
Preparation of modified mesoporous silica with calix[4]arene derivatives.

**Figure 2:**
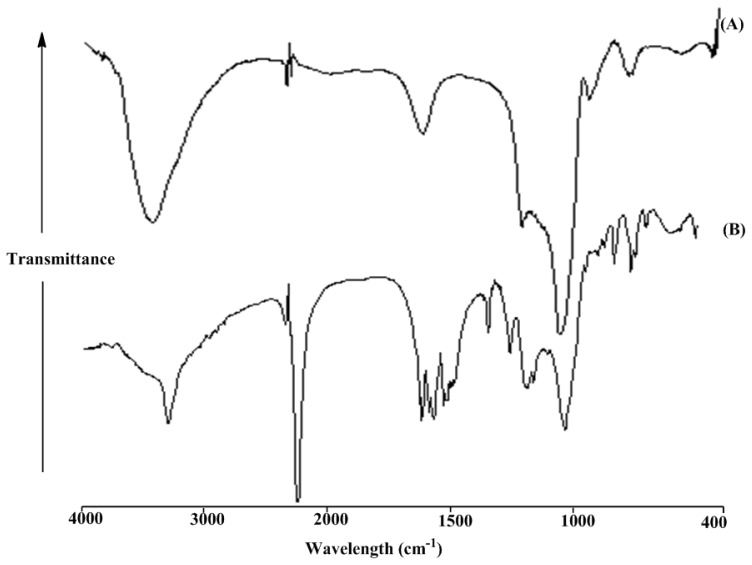
Fourier transform infrared spectroscopy (FTIR) spectra of MCM-41 (**A**) and MCM-toluene 2,4-di-iso-cyanate (TDI) (**B**).

**Figure 3:**
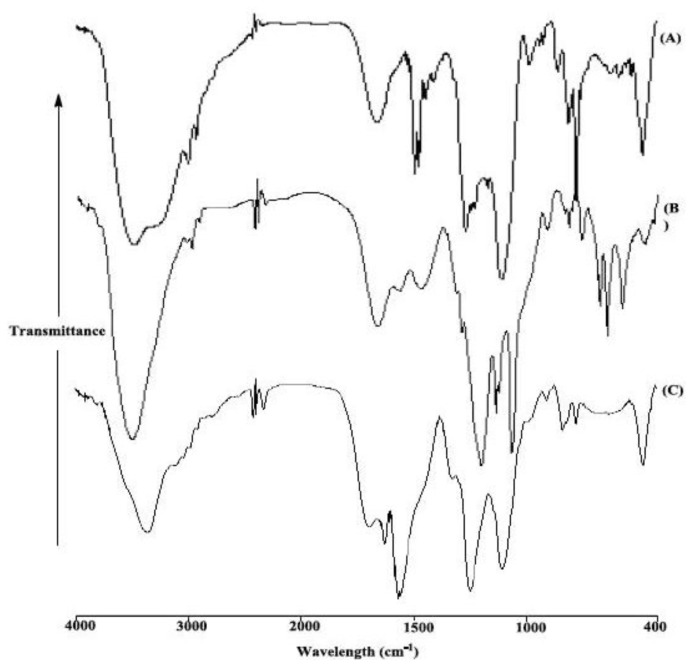
FTIR spectra of MC4 (**A**); MC4S (**B**) and MPC4 (**C**).

FTIR spectroscopic analysis provided the evidence that the mesoporous silica surface reaction proceeded as illustrated in Figure 1. Figures 2 and 3 show the FTIR spectra of activated mesoporous silica, M-TDI, C4-functionalized mesoporous silica MC4 (1), C4S-functionalized mesoporous silica MC4S (2) and PC4-functionalized mesoporous silicaMPC4 (3). The FTIR spectrum of unmodified mesoporous silica is relatively simple and well assigned [24]. The strong absorbance at 1102 cm^−1^ is attributed to the Si–O–Si stretch of silica, the absorbance at 1641 and 3455 cm^−1^ is assigned to the surface hydroxyl groups of mesoporous silica (Figure 2A). Addition of excess TDI to the mesoporous silica resulted in the incorporation of isocyanate functionalities on the surface of the mesoporous silica. This was evidenced by the appearance of a clearly discernible band at 2275 cm^−1^ corresponding to asymmetric stretching of the appended terminal isocyanate groups, and the appearance of an aromatic C–C stretch at 1549 cm^−1^ in the FTIR spectrum Figure 2B. The signals corresponding to the C=O and C–N stretches of the formed carbamate linkages between the mesoporous silica and the isocyanate functionality in compound mesoporous silica-TDI at 1647 cm^−1^ and 1197 cm^−1^ may be merged with the band of surface hydroxyl groups of mesoporous silica and Si–O–Si band, respectively.

The surface isocyanate functionalities could then be treated with calix[4]arene derivatives in dry toluene at 80 °C for 24 h. These functionalization reactions were again followed by FTIR spectroscopy to monitor the appearance and disappearance of some peaks. In detail, compared with the spectrum of M-TDI, MC4 1 (Figure 3A) present a strong band at 3423 cm^−1^ and its shoulder near 3198 cm^−1^, which correspond to the OH group of the mesoporous silica surface and the aromatic OH, respectively. The medium-intensity band at 1449 cm^−1^ corresponds to methylene bridges –CH_2_–. The band at 1078–1229 cm^−1^ of mesoporous silica-TDI spectra, which is referred to as Si–O–Si, was broadened with C_ar_–O stretching at 1241 cm^−1^. The spectra of MC4S 2 (Figure 3B) presents three main bands at 3449, 1446 and 1051 cm^−1^ assigned to the N–H and OH group of both hydroxides for the mesoporous silica and the C4S molecule, the weak absorption peak of methylene bridges –CH_2_– and the strong absorption peak of S–O which broadened the peak of Si–O–Si, respectively. In the case of MPC4 3 (Figure 3C), it can be seen that there were methyl (CH_3_) asymmetric stretching and symmetric vibrations at 2969 and 2862 cm^−1^, respectively, and bridges –CH_2_– at 1424cm^−1^. The bands at 807 and 755 cm^−1^ are related to aromatic torsion vibrations [25]. Meanwhile, the absorption at 2275 cm^−1^ in the spectra of MC4 1, MC4S 2, disappeared. This indicates that the unattached isocyano groups reacted with calix[4]arene derivatives, and calix[4]arene derivatives were successfully bonded on the surface of the M-TDI. But in the case of MPC4 3, the band at 2281 cm^−1^ corresponding to asymmetric stretching of an isocyanate group appears be due to the steric hindrances.

Elemental analysis provided further evidence of the successful modification of mesoporous silica. Table 1 gives the carbon, hydrogen, nitrogen and sulfur contents of M-TDI and mesoporous silica modified with calix[4]arene derivatives. It can be observed that the amount of carbon in mesoporous silica modified with calix[4]arene derivatives is higher compared to the carbon contents in M-TDI.

**Figure 4:**
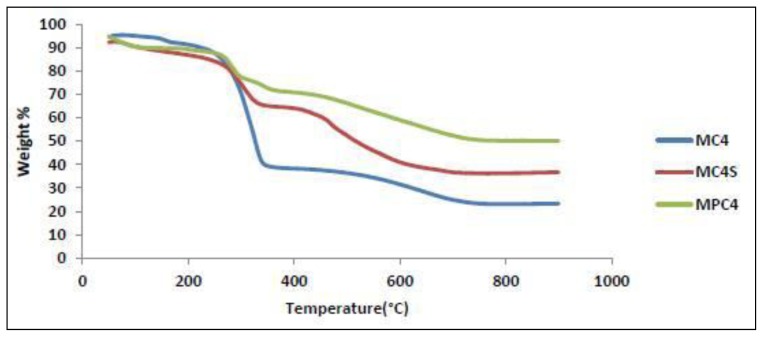
TGA-DTA analysis of MC4; MC4S and MPC4.

Thermogravimetric analysis of mesoporous silica, M-TDI, and M-TDI-calix[4]arene derivatives (1, 2 and 3) were determined (Figure 4). Mesoporous silica and some curves exhibited a stage of weight loss below 100 °C due to the loss of the adsorbed water. Above 100 °C, the curves were different. Mesoporous silica had hardly any weight loss above 100 °C. The weight-loss of M-TDI-calix[4]arene derivatives occurred at many regions (Table 2), and every curve exhibited a stage of weight loss that referred to the loss of a carbamate group. Based on these data, it is proven that the silica was successfully modified with calix[4]arene derivatives.

**Figure 5:**
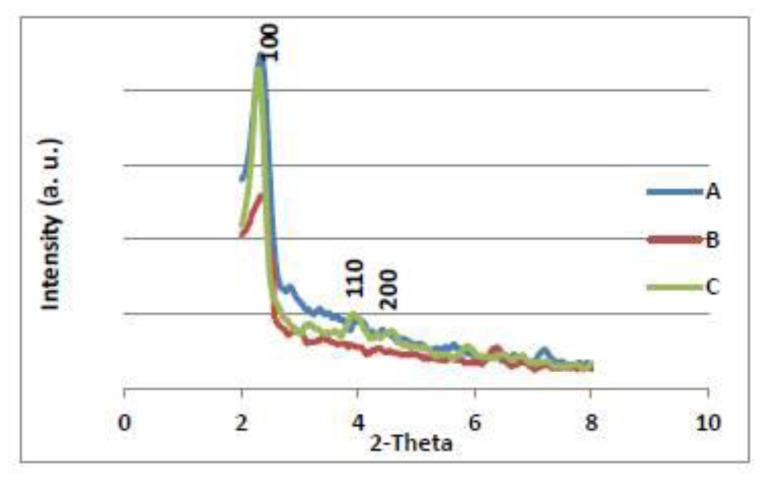
X-ray powder diffraction (XRD) analysis of MPC4 (**A**); MC4S (**B**) and MC4 (**C**).

Figure 5 shows the low angle range X-ray powder diffraction (XRD) patterns of the modified mesoporous silica with calix[4]arene derivatives MPC4 (A), MC4S (B) and MC4 (C). The pure MCM-41 starting material exhibits the peak patterns characteristic of mesopourous silica materials with a hexagonal symmetry [26]: three well resolved Bragg reflections for 2 h values between 2θ and 4,5θ, one very intense one due to the (100) reflection and two weaker peaks due to (110) and (200) reflections. Upon functionalization of MCM-41 with calix[4]arene derivatives, the XRD patterns of the samples show strong (100) peaks and smaller (110) and (200) peak intensities, suggesting that the modification process does not strongly affect the framework integrity of the ordered mesoporous MCM-41. The peaks (110) and (200) showed a decrease in overall intensities of XRD reflections of MCM-41 after calix[4]arene derivatives functionalization (Figure 5). This may be due to the difference of scattering contrast between the amorphous silicate framework and organic moieties, which are located inside the channels of MCM-41 [27,28].

**Figure 6:**
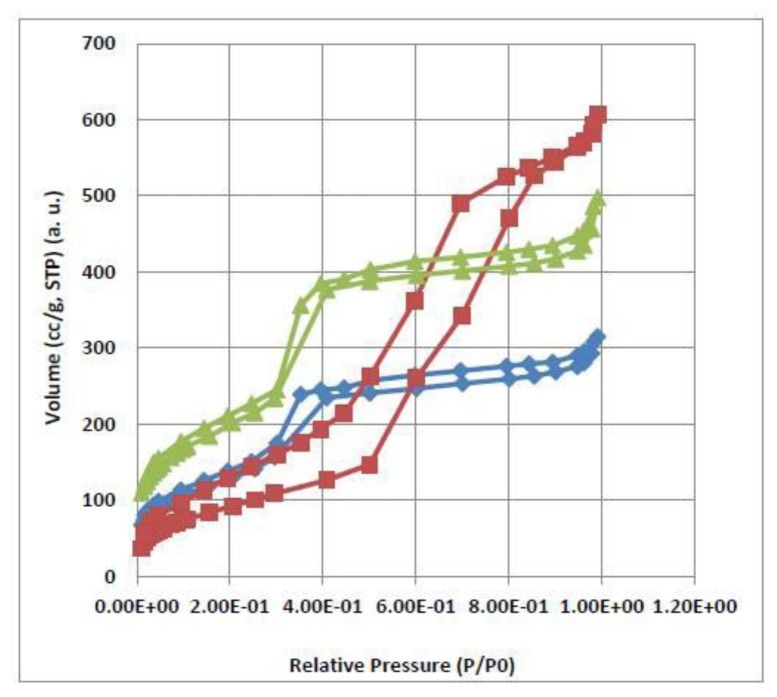
Nitrogen adsorption-desorption isotherms of (Δ) MC4; (**⋄**) MC4S; and (□) MPC4.

In order to further investigate the channel structure of these materials, the characterization of the nitrogen adsorption-desorption was also carried out. The corresponding isotherms are presented in Figure 6. They all exhibit the typical Type IV isotherms according to the International Union of Pure and Applied Chemistry (IUPAC) classification [29,30], corresponding to the characteristics of mesoporous materials with highly uniform size distributions. The structure data of these mesoporous materials (Brunauer-Emmett-Teller (BET) surface area, total pore volume, and pore diameter) are summarized in Table 3.

The grafted materials exhibit a broader pore diameter and display also a decrease in surface area and pore volume (Table 3). The decrease of the pore value and the broad distribution of pore size evidences that the calix[4]erene derivatives in the grafted mesoporous samples are mainly located on internal surfaces of the mesoporous materials [27,31,32].

## 3. Experimental Section

### 3.1. Materials

The chemicals used in this study are commercially available. Mesoporous silica (Aldrich, MO, USA, surface area 993 m^2^/g, average diameter of 2.9 nm) as silica sources. Calix[4]arene (C_28_H_24_O_4_, Acros, NJ, USA), *p*-*tert*-butylcalix[4]arene (C_44_H_56_O_4_, Fluka, MO, USA) were the organic modifier and toluene 2,4-di-iso-cyanate (TDI) (C_9_H_6_N_2_O_2_, Aldrich, Buches SG, Switzerland) was the organic linker. Triethylamine (C_6_H_15_N, SAFC, Steinheim, Germany) was used as catalyst. Toluene (Fisher, Loughborough, UK, dried before use by using molecular sieves), ethanol (Fisher, Loughborough, UK) and acetone (Fisher, Loughborough, UK) were used as solvents. Dichloromethane (CH_2_Cl_2_, Sigma Aldrich, Steinheim, Germany), chlorosulfonic acid (HSO_3_Cl, Merk, Hohenbrunn, Germany) and methanol for synthesis of *p*-sulfonatocalix[4]arene as described in the literature [33]. Water was purified using Milli-Q purification equipment.

### 3.2. Instrumentation

Fourier transform infrared spectra (FTIR) were recorded on a Perkin Elmer FTIR Spectrum RX1 ATR with a KBr pellet technique. Thermogravimetry (TG) and differential thermal analysis (DTA) were carried out from 50 °C to 900 °C at a heating rate of 20 °C/min in a nitrogen atmosphere using Perkin Elmer TGA 4000 analyzer. Elemental analyses were performed on a Perkin Elmer CHNS-2400 analyzer. Nitrogen adsorption-desorption experiments were carried out at 77.40 K on a Quantachrome Autosorb Automated Gas Sorption system. The Brunauer-Emmett-Teller (BET) surface area (SBET) was calculated from the linearity of the BET equation. The surface area, volume and pore diameter were calculated from the pore size distribution curves using the Density Functional Theory DFT method. The X-ray powder diffraction (XRD) patterns were obtained on a Bruker AXS D-8 Advance diffractometer using Cu K radiation (*λ* = 0.154056 nm) at 40 kV and 30 mA within the 2*θ* range of 2 to 100.

### 3.3. Synthesis Methods

#### 3.3.1. Introduction of Diisocyanate Functional Groups into the Mesoporous Surfaces

The functionalization of isocyanate groups onto MCM-41 surface was carried out using excess of TDI [34]. 5.0 g of silica (150 °C, 4 h) and 200 mL TDI (1.41 mol) (dried by molecular sieve 24 h) were mixed using a magnetic stirrer and the functionalization was undertaken in a dry nitrogen atmosphere at 80 °C for 4 h (Figure 1a). In order to remove all the substances physically adsorbed on the surface of the particles, the sample of mesoporous silica-TDI was separated by centrifugation and sequentially rinsed with toluene and acetone. The sample was marked as MCM-TDI.

#### 3.3.2. Determination of Isocyanate Groups of the Reaction System

The content of isocyanate groups of the reaction system was determined by titration. 200 mg of MCM-TDI sample and 20 mL of 0.1 mol/L di-*n*-butylamine in toluene were charged into a flask and the mixture was stirred at room temperature for 1 h. The unreacted di-n-butylamine was backtitrated with 0.1 mol/L HCl using bromophenol blue as an indicator. The content of isocyanate groups was calculated by the Equation:

$$\text{Isocyanate group }(\text{mmol}/\text{g})=0.1\;(V_{0}-V_{\text{s}})\;\frac{f}{w}$$

where *V*_0_ (mL) is the titer of 0.1 mol/L HCl for blank, *V*_s_ (mL) the titer of 0.1 mol/L HCl for the sample; *f*, the factor of 0.1 mol/L HCl; and *w*, the weight of the sample in gram(g).

#### 3.3.3. Grafting Modification of MCM-TDI with Calix[4]Arene Derivatives

Two millimoles (calculated from Section 3.3.2) of Calix[4]arene derivatives (Calix[4]arene C4, *p*-sulfonatocalix[4]arene C4S, para tert butyl Calix[4]arene PC4) was added into the MCM-TDI (1 gm) suspension with toluene (dried by molecular sieve 24 h). Subsequently, triethylamine was added and the reaction temperature was kept at 80 °C for 24 h under stirring. Then the particles were separated by centrifugation, washed with toluene and acetone and dried overnight. The samples were marked as MCM-TDI-calix[4]arene (MC4) 1, MCM-TDI-*p*-sulfonatocalix[4]arene (MC4S) 2, MCM-TDI-para-*tert*-butyl calix [4]arene (MPC4) 3. The basic scheme of the surface modification of mesoporous silica is shown in Figure 1.

## 4. Conclusions

In summary, we obtained the organic-inorganic mesoporous hybrid materials by linking the calix[4]arene derivatives to the functionalized ordered mesoporous MCM-41with toluene 2,4-di-iso-cyanate (TDI). The synthesis of MC4, MC4S and MPC4 provides a convenient approach of tailoring the surface properties of mesoporous silicates by organic functionalization, and the resulting materials of MC4, MC4S and MPC4 all retain the ordered mesoporous structures. Currently we are investigating the possibility of these materials as adsorbents for organometallic compound.

## Figures and Tables

**Table 1:** Results of elemental analysis for mesoporous silica-TDI; MC4 (1); MC4S (2) and MPC4 (3).

Sample	%C	%H	%N	%S
M-TDI	18.64	3.04	5.93	-
MC4	42.64	3.05	1.06	-
MC4S	33.31	4.16	3.97	4.04
MPC4	40.53	4.84	4.30	-

**Table 2:** Thermogravimetric analysis results of MC4 (1); MC4S (2) and MPC4 (3).

Sample	Region °C	Weight-loss %	Assignment
MC4	45–150	4.9	Moisture
	150–280	8.4	calix[4]arene
	280–380	21.4	Carbamate group and calix[4]arene
	380–800	32.49	calix[4]arene
MC4S	45–150	4.7	Moisture
	150–280	5.9	Carbamate group
	350–800	37.9	calix[4]arene sulfonate
MPC4	45–150	2.4	Moisture
	200–350	44.9	Carbamate group and para tert butyl calix[4]arene
	400–800	15.7	para tert butyl calix[4]arene

**Table 3:** Structural parameters of MCM-41; MC4 (1); MC4S (2) and MPC4 (3).

Sample	S_BET_ (m^2^/g)	V (cm^3^/g)	D (nm)
MCM-41	993	0.86	2.9
MC4	733	0.67	3.6
MC4S	452	0.43	3.8
MPC4	339	0.32	3.9
